# Characterizing Bacterial Cellulose Produced by*Komagataeibacter sucrofermentans* H-110 on Molasses Medium and Obtaining a Biocomposite Based on It for the Adsorption of Fluoride

**DOI:** 10.3390/polym13091422

**Published:** 2021-04-28

**Authors:** Viktor V. Revin, Alexander V. Dolganov, Elena V. Liyaskina, Natalia B. Nazarova, Anastasia V. Balandina, Anna A. Devyataeva, Vadim D. Revin

**Affiliations:** 1 Department of Biotechnology, Bioengineering and Biochemistry, National Research Ogarev Mordovia State University, 430005 Saransk, Russia; n.sapunowa2016@yandex.ru (N.B.N.); ania.devyataeva@yandex.ru (A.A.D.); Vadim.revin.16@mail.ru (V.D.R.); 2 Departmentof Inorganic and Analytical Chemistry, National Research Ogarev Mordovia State University, 430005 Saransk, Russia; dolganov_sasha@mail.ru (A.V.D.); balandina.av@yandex.ru (A.V.B.)

**Keywords:** bacterial cellulose, molasses, biocomposite, fluoride ions

## Abstract

Currently, there is an increased demand for biodegradable materials in society due to growing environmental problems. Special attention is paid to bacterial cellulose, which, due to its unique properties, has great prospects for obtaining functional materials for a wide range of applications, including adsorbents. In this regard, the aim of this study was to obtain a biocomposite material with adsorption properties in relation to fluoride ions based on bacterial cellulose using a highly productive strain of *Komagataeibacter sucrofermentans* H-110 on molasses medium. Films of bacterial cellulose were obtained. Their structure and properties were investigated by FTIR spectroscopy, NMR, atomic force microscopy, scanning electron microscopy, and X-ray structural analysis. The results show that the fiber thickness of the bacterial cellulose formed by the *K. sucrofermentans* H-110 strain on molasses medium was 60–90 nm. The degree of crystallinity of bacterial cellulose formed on the medium was higher than on standard Hestrin and Schramm medium and amounted to 83.02%. A new biocomposite material was obtained based on bacterial cellulose chemically immobilized on its surface using atomic-layer deposition of nanosized aluminum oxide films. The composite material has high sorption ability to remove fluoride ions from an aqueous medium. The maximum adsorption capacity of the composite is 80.1 mg/g (F/composite). The obtained composite material has the highest adsorption capacity of fluoride from water in comparison with other sorbents. The results prove the potential of bacterial cellulose-based biocomposites as highly effective sorbents for fluoride.

## 1. Introduction

Nowadays, there is a greater demand for biodegradable materials due to growing ecological concerns [1]. Natural materials based on plant (wood) cellulose, the most abundant organic biopolymer on earth, have long been used because they are widely availability, renewable, and biodegradable.

In recent years, there has been growing interest in bacterial cellulose (BC) [2]. There are interesting detailed reviews based on the production, characterization, and applications of BC [3,4,5,6,7,8,9].

BC is synthesized by bacteria such as Komagataeibacter (Gluconacetobacter), Agrobacterium, *Achromobacter*, Enterobacter, Rhizobium, *Pseudomonas*, *Salmonella*, Azotobacter, *Alcaligenes*, Sarcina, and Rhodococcus [10,11]. The most well-known producer of bacterial cellulose is the acetic acid bacterium Komagataeibacter xylinus.

BC is an ultrafine, nanofibrillar material with an exclusive combination of properties such as high purity, high crystallinity (up to 90%) and polymerization degree [7,12,13], high surface area, high flexibility and tensile strength [14,15], and high water-holding capacity (over 100 times its own weight) [16,17]. Due to its high purity, i.e., absence of lignin and hemicellulose, BC is considered to be a non-cytotoxic, non-genotoxic, and highly biocompatible material, attracting interest in diverse areas, particularly in medicine [9,18,19,20].

BC is nowadays considered a functional biomaterial with numerous applications in various fields, including biomedicine for tissue engineering [21,22,23], wound dressing [17,23,24,25,26], and controlled drug delivery systems [23]. BC can be used in dietetics as a carrier of additives for balanced nutrition, and in industrial electronics for the manufacture of acoustic diaphragms. It is a promising source for obtaining biocomposite materials [7,27].

BC meets many criteria to fit the profile of a highly efficient adsorbent [28]. The greatest advantage of modifying adsorbents with nanocellulose or nanocellulose-based adsorbents is the number of functional groups they provide due to the high surface area and functional group density. The numerous hydroxyl groups (or others, if chemically modified) on the nanocellulose result in a higher capacity of the target molecule to attach to the adsorbent [29,30,31,32,33]. Unlike other inorganic adsorbents, nanocellulose-based materials are also totally biodegradable, enabling both sustainability and biological use without side effects [34,35].

The presence of large amounts of fluoride in water is a pressing problem worldwide, and is caused by both geochemical processes and industrial production. Fluoride (F-) is essential for the growth of teeth and bones, but excessive F- intake causes dental and skeletal fluorosis [36]. Therefore, many countries and organizations have introduced strict criteria for fluoride control. The World Health Organization has set a strict limit for fluoride in drinking water of 1.5 mg/L, but despite growing concerns about this form of water pollution, the development of effective fluoride disinfection technologies remains a challenge.

Various physical and chemical methods exist that allow for removal of fluoride from water, such as chemical deposition, adsorption, reverse osmosis, and electrodialysis. Among the treatment options, adsorption is a competitive method compared to other processes. The innate simplicity of its design, relatively low cost, and industrial scalability make adsorption-based technologies attractive [37].

Currently, a search is underway for a new generation of adsorbents for use in purifying water from pollutants [38]. Different adsorbents for fluoride removal purposes have been generated from waste residue of the alum manufacturing process [39], untreated hydrated alumina (UHA) and thermally treated hydrated alumina (THA) [40], aluminum oxide–manganese oxide composite material [41], activated alumina [42] modified bauxite [43], non-conventional adsorbents (fly ash, modified neem bark powder and fish scale biochar) [44], nano-magnetically modified activated carbon [45], porous hydroxyapatite ceramics [46], magnesia-hydroxyapatite adsorbent [47], nanoscale aluminum oxide hydroxide (AlOOH), and natural zeolite [48]. Bio-based composites have been investigated by researchers as promising adsorbents. Several works were carried out using bamboo-carbon as an adsorbent for fluoride removal [49]. Activated bamboo charcoal impregnated with iron-aluminum [50], and coconut fiber ash impregnated with aluminum have also been investigated in fluoride removal studies [51]. However, from the wide variety of sorbents presented for the removal of fluoride ions from water, all of them are ineffective.

Therefore, the aim of this work was to obtain a more efficient bio-based adsorbent to fluoride ions. Since BC is a promising source for obtaining bio-based biocomposite materials, another important task was cost-effective production of BC using a highly productive strain of *Komagataeibacter sucrofermentans* H-110 and by-product of sugar industry. Moreover, the structure and properties of BC produced from molasses were analyzed by FT-IR, NMR, AFM, SEM, and XRD.

## 2. Materials and Methods

### 2.1. Chemicals and Materials

The reagents used—Alizarin complexone, lanthanum nitrate 6-aqueous, sodium acetate 3-aqueous, sodium hydroxide, acetic acid, and FIXANAL ampoules of hydrochloric and nitric acid—Were obtained commercially (Sigma-Aldrich^®^, Saint Louis, MO, USA). Deionized water was used in the experiments.

### 2.2. BC Preparation

BC was prepared in a static culture medium by *Komagataeibacter* *sucrofermentans* H-110, which was isolated from Kombucha tea and identified by sequencing the amplified product of 16S rRNA [11]. A strain was deposited in the Russian National Collection of Industrial Microorganisms (VKPM) (accession no.: B-11267). The molasses medium for BC production contained sugar beet molasses, 45.0 g/L, pH 4.5, and Hestrin and Schramm (HS) medium (g/L): glucose (20), peptone (5), yeast extract (5), citric acid (1.15), and disodium hydrogen phosphate (2.7), pH 6.0. Culture medium was autoclaved for 20 min at 120 °C. The medium was inoculated with 10% (*v*/*v*) inoculum. To prepare the inoculum, *K. Sucrofermentans* H-110 from an agar plate was transferred aseptically into a 250 mL Erlenmeyer flask containing 100 mL of culture medium and incubated on a shaker incubator (Model ES-20/60, BIOSAN, Latvia) at 28 °C for 24 h at 250 g. BC was produced in static conditions at 28 °C for 5 days. After incubation, BC was collected, washed thoroughly with deionized water to remove medium components, and treated with 1% (*w*/*v*) sodium hydroxide solution for 1 h at 80 °C to eliminate bacterial cells. Further, BC was rinsed extensively with 6% (*v*/*v*) acetic acid and then deionized water until pH became neutral. The purified BC was dried to constant weight at 60 °C. BC production is reported as gram dry weight of cellulose per liter of medium (g/L).

### 2.3. Atomic Force Microscopy (AFM)

The surface morphology of BC was studied by contact atomic force microscopy (AFM) using an SPM 9600 microscope (Shimadzu, Japan). BC samples were air-dried on glass slides in the form of a thin film. A silicon nitride cantilever with a pyramidal tip that had a nominal radius of 2 nm was used. Scan rates ranged from 0.6 to 1.0 Hz/s. Image resolution of 256 × 256 points was set.

### 2.4. Scanning Electron Microscopy (SEM)

Scanning electron microscopy (SEM) of the BC and BC modified with aluminum oxide was performed using a Quanta 200 I 3D FEI scanning electron microscope (Hillsboro, OR, USA).

### 2.5. Fourier Transform Infrared (FT-IR) Spectroscopy

BC was freeze-dried and crushed into powder form, mixed with potassium bromide, and pressed into small tablets that were subjected to Fourier transform infrared spectroscopy (FT-IR) using an IRPrestige-21 spectrophotometer (Shimadzu, Japan) in absorption mode. For each sample, 32 scans at 4 cm^−1^ resolution at wave numbers ranging from 4000 to 400 cm^−1^ were collected.

### 2.6. X-ray Diffraction (XRD)

X-ray diffraction (XRD) measurement was carried out to analyze changes in crystallinity of the produced BC by an Empyrean x-ray diffractometer (PANalytical, Lelyweg, Netherlands) in filtered radiation of the copper anode (λ = 0.15418 nm, 40 kV, 30 mA) within an angular range (2θ) from 10 to 60°. A two-coordinate Pixcel 3D detector working in linear scanning mode (255 pixels on a strip) with a resolution of 0.013°/strip was also used. The samples were freeze-dried using a FreeZone Freeze Dry System (Labconco, Mostowa, Kędzierzyn-Koźle, Poland). The crystallinity index (CrI) was calculated from the ratio of the height of the 002 peak (I_002_, 2θ = 22.5°) and the height of the minimum (Iam) between the 002 and 110 peaks (Iam, 2θ = 18°) (Equation (1)).

CrI (%) = [(I_002_ − I_am_)/I_002_] × 100% (1)

### 2.7. ^13^C NMR Spectroscopy

^13^C NMR spectra were recorded on a JEOL JNM-ECX400 spectrometer (9.39 T, 100.5 MHz) in the solid phase at room temperature using the cross-polarization technique (CPMAS) with a rotation speed of 10 kHz in 7 mm zirconia rotors. The magic angle spinning (MAS) of the sample was determined at a rotation speed of 10 kHz. All MAS experiments were performed at room temperature; proton decoupling was performed using two-pulse phase modulation (TPPM). When registering the ^13^C MAS NMR spectra, rotary synchronization of the rapid spin echo (RSE) sequence or single pulse (SP) excitation at a Larmor frequency of 100.6 MHz was used. To optimize the spectrum registration process, the relaxation time of carbon nuclei was selected. The pulse duration for the 90° angle was 6 ms, and for 180°, it was 12 ms, and the total number of scans was 256. The spectra were processed using the ACD/NMR Processor Academic Edition, Ver. 12.01.

### 2.8. Sorbent Preparation: Deposition of Aluminum Oxide on Surface of BC UsingAtomic Layer Deposition

The sorbent was BC, on which films of Al_2_O_3_ of various thicknesses were deposited using atomic layer deposition (ALD) technology. The thin film deposition using the ALD method was carried out as follows: The substrate in a vacuum chamber at operating temperature was alternately exposed to two reagents (precursors) in a vapor state. Precursors reacting only on the substrate surface formed a monolayer of a thin-film compound. The thickness of the applied layers was determined using previously obtained calibration lines. When the film of aluminum oxide (Al_2_O_3_) was deposited, trimethylaluminum (Al (CH_3_)_3_ (TMA)) and water (H_2_O) were used as precursors; the reactor temperature was 100° C. This process is well studied: film growth occurs due to self-limited reactions, and the aluminum containing the precursor is highly reactive.

The process can be roughly divided into 4 stages: (1) Introducing trimethylaluminum vapors into the chamber. On the surface of the substrate, trimethylaluminum is bound to the surface, losing one of its methyl groups, and two methyl groups bound to the aluminum atom become active surface groups. (2) Purging the chamber with a carrier gas to remove the precursor residues and the reaction products. After blowing, a precursor bound to the surface remains on the surface of the substrate. (3) Introducing water vapor into the chamber. Water molecules instantly react with the surface monolayer, forming an oxide film and releasing methane as a volatile reaction product. After this stage, hydroxyl groups become active surface groups. (4) Purging the chamber with a carrier gas to remove residual water vapor and reaction products. Upon the subsequent introduction of the first precursor (in our case, trimethylaluminum), it reacts with surface hydroxyl groups, starting a new cycle of the ALD process, resulting in a thin film with active surface groups participating in the next half-cycle.

In simplified form, the ALD process can be written as follows: Al (CH_3_)_3_ + OH * = Al (CH_3_)_2_ * OH + CH_4_, Al (CH_3_)_2_ * OH + H_2_O = Al_2_O_3_ * OH + 2CH_4_, etc., where asterisks denote surface groups of the substrate.

### 2.9. Adsorption Studies

Adsorption experiments were carried out in stationary mode. Samples of a film of bacterial cellulose modified with aluminum oxide weighing 0.01 to 0.02 g were introduced into a solution of fluoride ions with a concentration of 5 mg/L and stirred for 1 h at a speed of 200 rpm. Upon reaching equilibrium, the mass concentration of fluoride ions was measured photometrically on a Shimadzu UV-1800 UV-visible spectrophotometer.

Using the method of atomic absorption spectroscopy (AAS), the content (wt.%) of alumina deposited on cellulose was determined. The content ranged from 41.40 to 66.94%, depending on the sorbent layer. Each experiment was performed 3 times under the same conditions, and the standard deviation of measurements was within ±2%. Using the AAS method, solutions after sorption were also studied in order to investigate the process of ion leaching from the sorbent into the solution.

Further, the calculation of adsorption capacity was carried out in terms of the total weight of the sorbent and separately for aluminum oxide according to Equation (2):

(2) $$A=\frac{C_{0}-C}{m}\cdot V,$$

where *A* is the adsorption capacity of the sorbent (mg/g), *C*_0_ is the initial concentration of fluoride ions in the solution (mg/L), *C* is the equilibrium concentration of fluoride ions in the solution after sorption (mg/L), *m* is the total mass of the sorbent and its mass in terms of Al_2_O_3_ (g), and *V* is the volume of the solution in which sorption is carried out (L).

A model solution of fluoride ions of a certain concentration was prepared by diluting a standard sample.

### 2.10. Statistical Analysis

All presented data are averages of at least 3 runs of experiments, performed with 3 to 6 replicates of the mean. Standard deviations of the mean were calculated using Microsoft Excel 2013 (Microsoft Corporation, Redmond, WA, USA). The obtained data were statistically analyzed by Student’s *t*-test, two-sample assuming equal variances. The differences were considered significant at the level of *p* < 0.05.

## 3. Results and Discussion

### 3.1. Characterization of BC Produced by K. sucrofermentans H-110 on Molasses Medium

**Figure 1:**
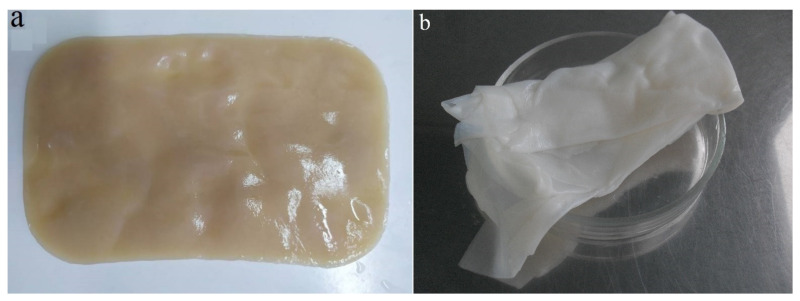
Gel film of BC produced by *K. sucrofermentans* H-110 using (**a**) molasses medium and (**b**) after purification.

It is recognized that properties of BC (e.g., crystallinity, dimensions, etc.) depend on the culturing conditions and/or bacteria employed [52,53]. The macrostructural morphology of BC varies depending on the different culture methods [6]. Cultivation of *K.* *sucrofermentans* H-110 bacteria in static conditions for 5 days on the surface of the molasses medium formed a BC gel film (Figure 1a). After treatment, the gel film became colorless and transparent (Figure 1b).

One of the main disadvantages of industrial BC production is the high cost of manufacture, mainly due to the commercial raw materials used in the formulation of fermentation media and the low productivity achieved during fermentation. Traditional carbon sources in BC production are glucose, fructose, and glycerol, which significantly increase the expense, representing ~30% of the total BC production cost [54,55]. To reduce the cost, alternative natural carbon sources are utilized, such as waste substrate from various industrial sectors (e.g., the food industry) [5,56,57].

Earlier in our studies, it was shown that *K. sucrofermentans* B-11267 forms a great amount of BC using acidic food industry by-products such as thin stillage and whey [52].

In our present work, we studied the formation of BC in medium containing sugar beet molasses at a concentration of 45 g/L with a sucrose content of 25 g/L. Studies have shown that the greatest accumulation of bacterial cellulose (2.9 ± 0.1 g/L) occurs on molasses medium with 5 days of cultivation, which is almost two times higher than on standard Hestrin and Schramm medium (1.6 ± 0.1g/L) (Table 1).

Machado et al. (2018) showed that the introduction of sugarcane molasses as nutrient source promoted a significant cost reduction of culture media by up to 20.06% relating to the conventional HS medium and, consequently, the cost of BC production [58]. BC yield was 1.9 g/L under static conditions for 5 days.

Molasses is a side product of the final stage of crystallization in sugar production and is one of the most economical carbon sources for the microbiological industry. It contains up to 80% of dry matter, of which sucrose makes up ~48%. Molasses also contains amino acids, organic acids and their salts, betaine, mineral compounds, and vitamins.

**Figure 2:**
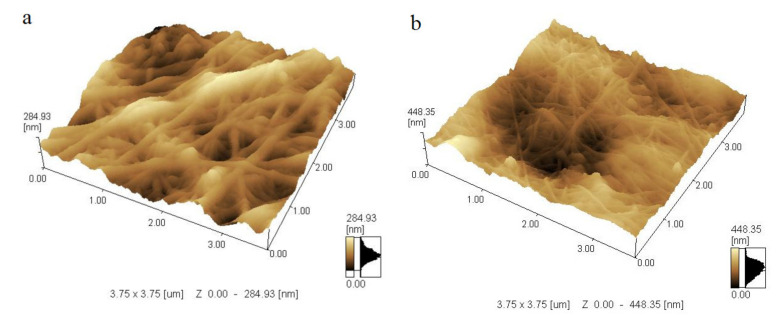
AFM images of cellulose microfibrils secreted into (**a**) HS medium and (**b**) molasses medium.

To study the microscopic details of the biopolymer, atomic force microscopy (AFM) was used. Figure 2 shows the micromorphology of BC, which exhibited a nanoporous three-dimensional network structure with a random arrangement of ribbon-shaped fibrils.The thickness of BC fibrils formed on standard HS medium and molasses medium averaged 50–90 nm.

**Figure 3:**
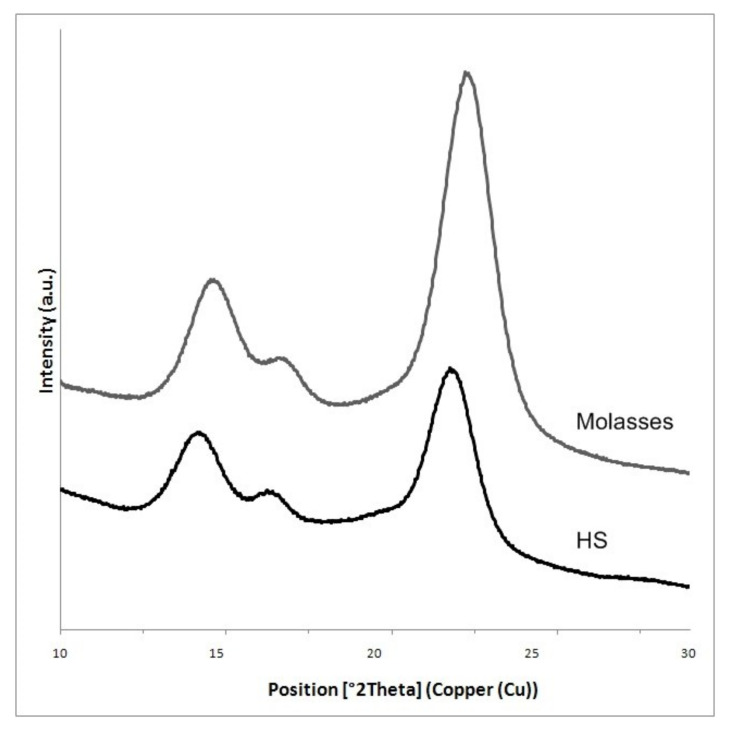
XRD patterns of BC obtained from HS medium and molasses.

In order to evaluate the crystalline structure and the change in crystallinity of BC produced from different culture media, X-ray diffraction was used, and the X-ray patterns of BC are shown in Figure 3. The obtained X-ray patterns show cellulose with the same chemical structure but different degrees of crystallinity (Table 1). The diffraction diagrams produced by all BC samples show three main peaks at ^o^2 Theta 14.4°, 16.8°, and 22.5°, corresponding to crystallographic planes of (100), (010), and (110), respectively. The intensity of the 100 reflection was larger than that of the 010 when the film was parallel to the X-ray beam, and the effect was reversed in the perpendicular orientation. This reveals a strong uniplanarity due to the fact that the cellulose ribbons are preferentially oriented parallel to the film surface during drying.

The results showed that on the medium with molasses, the degree of crystallinity of BC (83.02%) was larger than that in the standard HS medium (79.7%) (Table 1, Figure 3).

**Figure 4:**
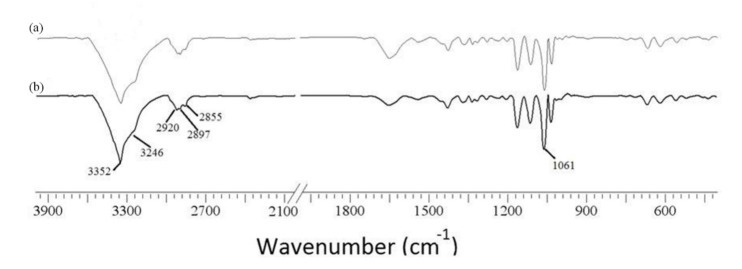
FT-IR spectra of BC produced from (**a**) HS medium and (**b**) molasses.

To study the chemical structure of BC, we analyzed FT-IR spectra at wavelengths ranging from 400 to 4000 cm^–1^. As shown in Figure 4, the functional groups of BC samples obtained from fermentation using molasses and HS medium were almost the same. The characteristic bands of cellulose (type I) appeared at 3352 and 3246 cm^–1^ for the stretching vibration of hydroxyl groups (–OH), at 2897 cm^–1^ for the asymmetric stretching vibration of methylene bridge (–CH_2_–), at 2855 cm^–1^ for the symmetric stretching vibration of methyl (–CH_3_), and at 1061 cm^–1^ for the C–O–C and C–O–H stretching vibrations of the sugar ring. The signals near 3240 and 750 cm^–1^ were assigned to the triclinic Iα allomorph, and the signals near 3270 and 710 cm^–1^ were assigned to the monoclinic Iβ form. Therefore, the synthesized cellulose contained both allomorphs.

**Figure 5:**
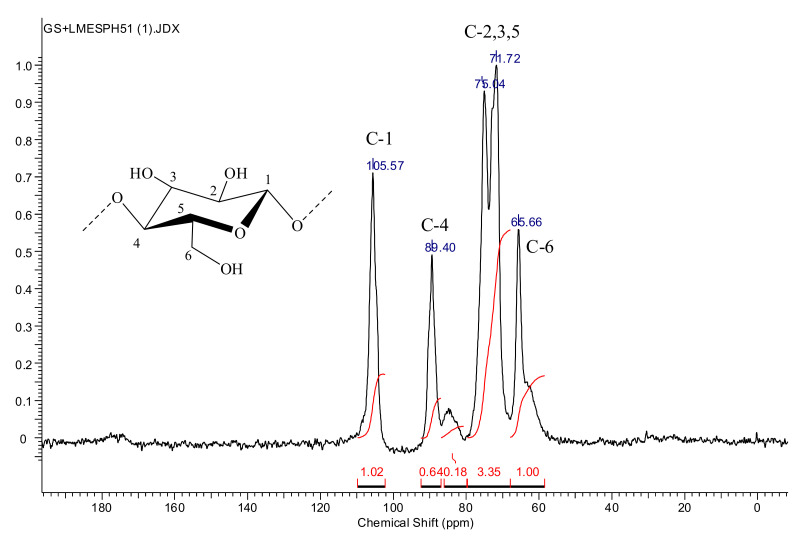
^13^C NMR spectra of BC.

^13^C NMR spectra of BC are shown in Figure 5.

In the spectrum of cellulose (Figure 5), four groups of signals are traced. According to Mori et al. [59], the region of 105–106 ppm corresponds to signals of acetal carbon atoms C-1, 80–90 ppm to signals of carbon atoms C-4, (89–90 ppm to crystalline, at 89.4 ppm, ~80%), and 84–85 ppm to amorphous form (~20%). The region of 70–80 ppm contains the signals of the C-2, C-3, and C-5 atoms of the pyranose ring, and the 60–68 ppm region contains the signals of the C-6 atom.

### 3.2. Surface Modification of BC with Aluminum Oxide Using Atomic Layer Deposition

**Figure 6:**
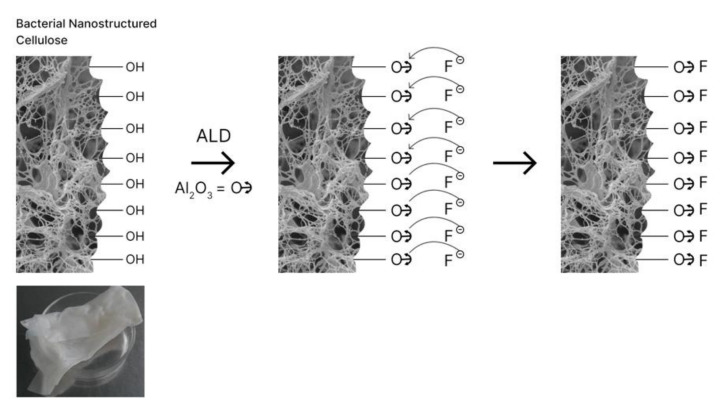
The scheme of the preparation process of biocomposites based on BC for the adsorption of fluoride.

BC has a unique combination of properties: a high degree of crystallinity with a large amount of “anchor” hydroxyl groups on the surface make it possible to modify the film surface ALD methods by forming a durable nanoscale layer of Al_2_O_3_ for further study as a sorbent to remove fluoride ions from water (Figure 6).

**Figure 7:**
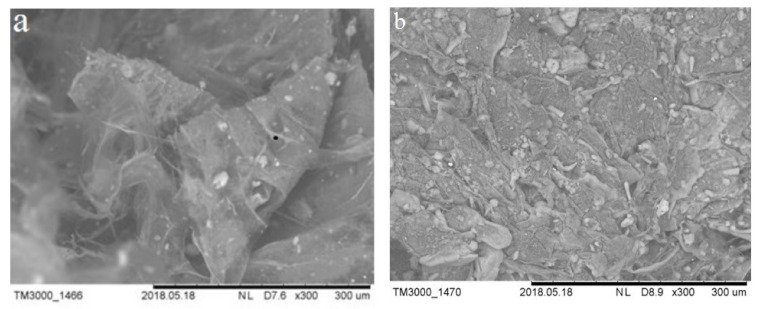
(**a**) SEM image of BC at 300 magnification; (**b**) SEM image of BC modified with aluminum oxide at 300 magnification.

SEM images of BC and BC modified with aluminum oxide are shown in Figure 7.

As you can see in Figure 7, aluminum oxide forms a dense film on the cellulose surface.

**Figure 8:**
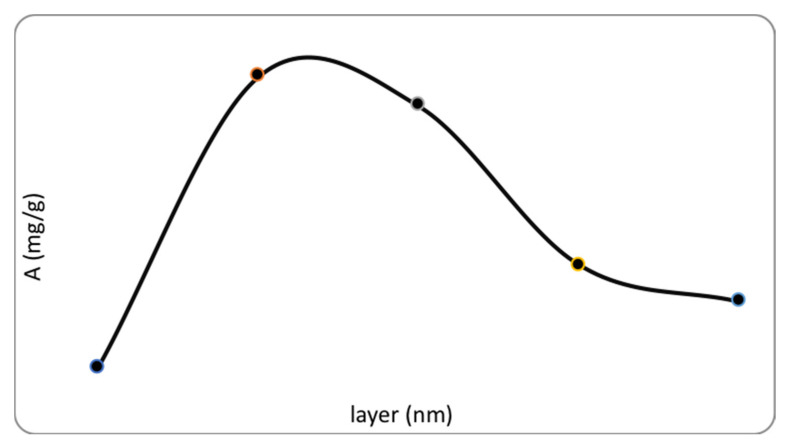
Dependence of adsorption capacity on thickness of aluminum oxide layer on biopolymer with pH value of solution.

To determine the optimal conditions for the adsorption of fluoride ion sorbents with different thicknesses of the Al_2_O_3_ layer, 50, 100, 150, and 200 nm were studied. Surprisingly, it turned out that the maximum adsorption capacity was achieved at an aluminum oxide layer thickness of 50 nm, and the minimum value at a thickness of 200 nm (Figure 8, Table 2). Most likely, with increased film thickness, formation of the alpha-Al_2_O_3_ phase occurs, which leads to “vacancies” for binding with fluoride ions. It should be noted that a 50 nm thick aluminum oxide film is X-ray amorphous, which, as it turned out, became the main criterion for an efficient sorption process. To calculate the adsorption capacity of the sorbent, samples weighing 0.01 to 0.02 g were introduced into a solution of fluoride ions at a concentration of 5 mg/L and stirred for 1 h at a speed of 200 rpm. Previously, using atomic absorption spectroscopy, the content of alumina deposited on the cellulose in mass% was determined. Its content ranged from 41.40 to 66.94%, depending on the sorbent layer. Further, the calculation of adsorption capacity was carried out in terms of aluminum oxide according to Equation (3):

(3) $$A=\frac{C_{0}-C}{m}\cdot V$$

where *A* is the adsorption capacity of the sorbent (mg/g); *C*_0_ is the initial concentration of fluoride ions (mg/L); *C* is the concentration of fluoride ions after sorption (mg/L); *m* is the mass of the sorbent in terms of Al_2_O_3_ (g), and *V* is the volume of the sorbed solution (L).

Table 2 shows the values of adsorption capacity depending on the thickness of the sorbent layer.

As can be seen from the data presented in Table 2, the adsorption capacity at a sorbent thickness of 50 nm reaches 80.1 mg/g, which is a reported characteristic among all described sorbents.

The obtained composite material based on BC modified with an alumina nanolayer using ALD technology has the highest adsorption capacity of fluorine from water (80.1 mg/g) in comparison with other sorbents, such as pine sawdust modified with aluminum (up to 4 mg/g) [60], modified bauxite (10 mg/L) [43], a sorbent of mixed oxides Mg-Al synthesized using fluff of the chinar tree (FCT) (55 mg/g) [61].

### 3.3. Effect of Solution pH on Fluorine Adsorption

**Figure 9:**
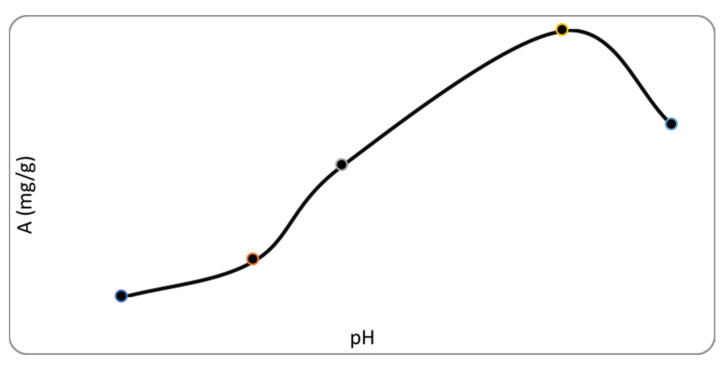
Dependence of adsorption capacity on pH.

Further, all experiments were carried out with a sorbent with a thickness of 50 nm. To study the effect of the acidity of the medium on the sorption process, studies were carried out at different pH values, 1.5–8. Figure 9 shows the adsorption of fluoride ions on a biopolymer as a function of solution pH. As can be seen from Figure 9, with pH increased to 7, the adsorption capacity increased, and then dropped sharply at pH 8. It is logical to assume that increased basicity of the solution would lead to the emergence of competition between fluoride and hydroxide ions to bind to the sorbent due to the interaction mechanism of Lewis acid–Lewis base. In strongly acidic solutions, most of the fluoride ions are in an undissociated form, which reduces their ability to bind to vacant sorbent centers.

### 3.4. Sorption Kinetics

**Figure 10:**
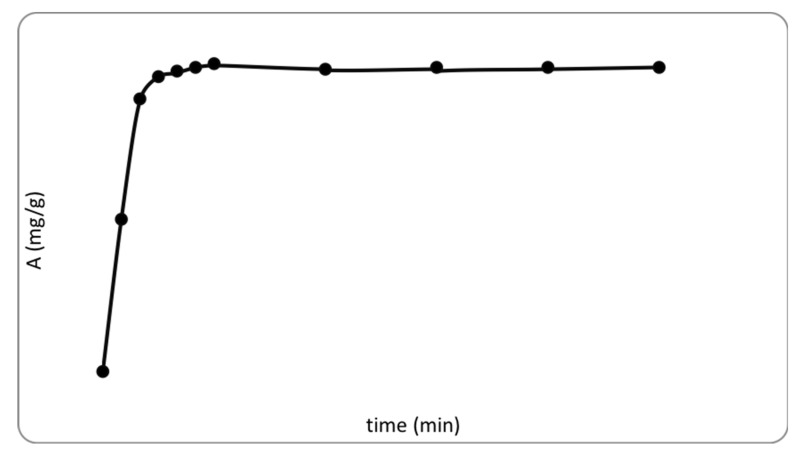
Sorption of fluoride ions from aqueous solution depending on time.

Studying the kinetics of sorption makes it possible to establish the rate of attainment of equilibrium and the mechanism of interaction of fluorine ions with the sorbent. As seen in Figure 8, up to 60 min, the adsorption capacity increased significantly, and after 60 min, a slight decrease occurred along with a state of equilibrium. This is explained by the fact that up to 60 min, the water-insoluble AlF_3_ complex is actively formed, which remains on the surface of the sorbent. A slight decrease in capacity after 60 min is probably due to a slight dissolution of AlF_3_ through its transition into solution in the form after reaching equilibrium, forming a complex ion AlF_6_^3−^, which passes into the solution (Figure 10).

It is known from the literature that the mechanism of ion sorption has a complex multistage nature and it is difficult to consider all stages of the process; therefore, when studying it, models based on the principles of determining the limiting stage of sorption are widely used (Tran et al., 2017). The criterion for determining the stage that limits the rate of ion sorption is the observation of a linear dependence of ln (1 − *F*) on t for external diffusion and *Г_t_* on *t^1/2^* for intradiffusion. For the external diffusion process, when the stage that controls the rate of the entire process is diffusion in a fixed film of solution around the sorbent grain, the kinetic curve is described by Equation (4):

ln (*1* − *F*) = −*y·t*, (4)

where *t* is time (min); *y* is some constant value for the given conditions, and *F* is the degree of reaching equilibrium, calculated as *F* = at/ap, where at and ap are the amount of sorbed substance at time *t* (mmol/g) and in equilibrium (mmol/g), respectively. For a process in which the limiting stage is internal diffusion, the observation of a straight-line dependence in the coordinates *Г*_t_ on *t*^1/2^ serves, the kinetic curve is described by Equation (5):

*Г_t_* = *K_d_·t^1/2^* + *A*, (5)

where *Г_t_* is the amount of *F*^−^ per unit mass of the sorbent (mmol/g); *K_d_* is the rate constant of internal diffusion (mmol∙g^–1^∙min^–0.5^); *t* is time (min), and *A* is a value proportional to the thickness of the film surrounding the sorbent grain (segment cut off by continuation of the direct dependence *Гt* = *f* (*t*) on the ordinate axis).

**Figure 11:**
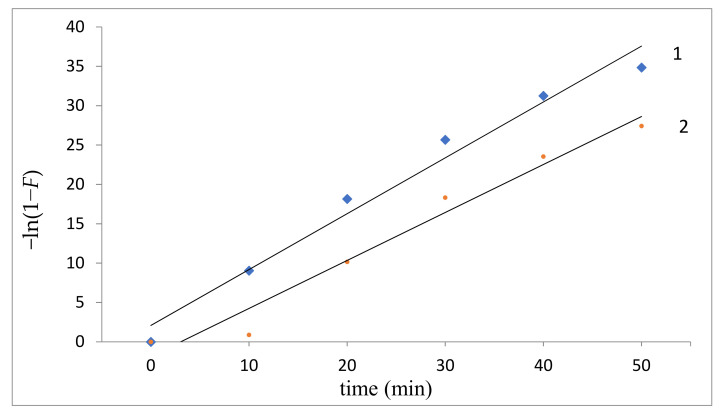
Dependence of −ln (1 − *F*) on sorption time of fluoride ions (1-sorbent layer thickness 50 nm; 2-sorbent layer thickness 100 nm).

To determine the limiting stage of the studied process, models and samples with thicknesses of 50 and 100 nm were used. Two-layer thicknesses were used to study the effect of sorbent layer thickness on the kinetics of fluoride ion sorption: sample 1, 50 nm; sample 2, 100 nm. In the case of sample 2 with an Al_2_O_3_ layer of 100 nm (Figure 11, curve 2), only in the initial portion of the –ln (1 − *F*) dependence on *t* is the rectilinear character of the function −ln (1 − *F*) = *f* (*t*), which corresponds to the course of the external diffusion mechanism process. Subsequently, the straightness of the kinetic curve is violated, which indicates a change in the sorption mechanism to intradiffusion. Thus, the obtained regularity shows the classic mixed-diffusion sorption mechanism, that is, diffusion of sorbate from the solution to the surface of the sorbent through the film and diffusion of the sorbate in the sorbent grain. As shown in Figure 11 (curve 1), for a sample with an Al_2_O_3_layer of 50 nm, practically over the entire sorption range, a rectilinear character of the −ln (1 − *F*) = *f* (*t*) function is observed, which indicates sorption of fluoride ions by the external diffusion mechanism by sample 1.

**Figure 12:**
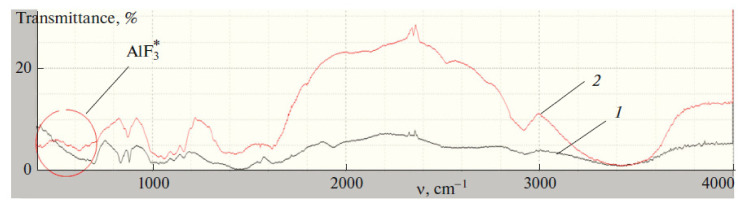
Change in IR spectrum during sorption of fluoride ions: (1) IR spectrum of original sorbent, and (2) after sorption.

The obtained dependencies were used to determine the kinetic parameters (rate constant of internal diffusion *K_d_*) characterizing the internal diffusion of fluoride ions by samples 1 and 2: *K_d_*_1_ = 0.22; *K_d_*_2_ = 0.13. The rate constant of internal diffusion *K_d_*, found from the tangent of the angle of inclination *Г_t_* from *t^1/2k^* to the abscissa, show a greater internal diffusion rate for sample 1 than sample 2. The isotherms of fluoride ion sorption by samples 1 and 2 in both cases corresponded to the isotherms of the Langmuir model. The initial straight-line portions of the curves show that adsorption was localized on separate adsorption centers, each of which interacted with only one adsorbate molecule, forming a monomolecular layer. Areas on the isotherms with high concentrations correspond to the sorbent surface completely saturated with sorbate. The middle sections of the sorption isotherms correspond to intermediate degrees of filling the sorbent surface. The occurrence of the ion-exchange mechanism of sorption of fluoride ions by samples 1 and 2 is confirmed by the data of IR spectroscopic analysis (Figure 12).

After sorption, the IR spectra of the samples exhibited absorption bands in the range of 735 and 740 cm^–1^, which are related to the antisymmetric stretching vibrations of the Al–F bond.

## 4. Conclusions

Bacterial cellulose was obtained using the strain we isolated, and its properties were characterized using a wide range of physical methods. To reduce the cost of obtaining bacterial cellulose, a by-product of sugar production was used. Studies have shown that the greatest accumulation of bacterial cellulose (2.9 g/L) occurs on molasses medium with 5 days of cultivation, which is almost two times higher than on standard Hestrin and Schramm medium (1.6 g/L). It has been shown that the infrared spectra of bacterial cellulose obtained in a variety of environments have similar characteristics, but we found differences in the crystallinity of the resulting biopolymer.

The sorption of fluoride ions by a material based on bacterial cellulose on which a nanolayer of aluminum oxide was deposited using atomic-layer deposition was studied. The results show that the maximum adsorption of fluorine occurred at a minimum sorbent layer thickness of 50 nm. The main parameters for efficient sorption of fluoride ions and water were found. Isotherms of fluoride ion adsorption were obtained based on the calculation of the kinetic parameters of the process, to determine the limiting stage of sorption. High sorption capacity of the composite material is shown in comparison with the described sorbents.

Thus, the results of this study present an excellent viewpoint to produce bacterial cellulose-based highly effective sorbents for fluoride using food industry by-products. These results can be helpful in the further development of bacterial cellulose-based materials as well as other new products for a wide range of applications, including highly efficient adsorbents.

## Figures and Tables

**Table 1:** BC yield, width of cellulose microfibrils by AFM and crystallinity by X-ray diffraction. HS—Hestrin and Schramm.

Medium	BC (g/L)	Width (nm)	Crystallinity (%)
HS	1.6 ± 0.1	60–90	79.7
Molasses medium	2.9 ± 0.1	60–90	83.02

**Table 2:** Influence of sorbent layer thickness on sorption capacity.

Sorbent Layer Thickness (nm)	A (mg/g)
50	80.1
100	66.3
150	14.7
200	9.8

## Data Availability

Not applicable.
